# Effects of imiglucerase on the growth and metabolism of Gaucher disease type I patients: a systematic review

**DOI:** 10.1186/1743-7075-10-34

**Published:** 2013-04-09

**Authors:** Divair Doneda, Cristina B Netto, Cileide C Moulin, Ida Vanessa D Schwartz

**Affiliations:** 1 Post Graduation Program in Medical Sciences, Universidade Federal do Rio Grande do Sul (UFRGS), Porto Alegre, Brazil; 2 Laboratory L.T.D, COMGRAD Nutrition, Medical School, UFRGS, Porto Alegre, Brazil; 3 Medical Genetics Service, Hospital de Clínicas de Porto Alegre (HCPA), Rua Ramiro Barcelos, 2350, Porto Alegre, RS, CEP: 90035-903, Brazil; 4 Centre for Studies in Food and Nutrition (CESAN), HCPA/UFRGS, Porto Alegre, Brazil; 5 Laboratory B.R.A.I.N, HCPA/UFRGS, Porto Alegre, Brazil; 6 Genetics Department, UFRGS, Porto Alegre, Brazil

**Keywords:** Gaucher Disease, Imiglucerase, Growth, Metabolism

## Abstract

**Background:**

Gaucher disease (GD) type I is the most common type of GD. Its main clinical manifestations are hepatosplenomegaly as well as bone and hematological abnormalities. The objective of the present study was to perform a literature review on the growth and metabolism of GD type I patients.

**Methods:**

We searched Pubmed and Scielo.br databases with predetermined study limits: case series (n≥5), clinical trials, systematic reviews, and meta-analyses, and enzyme replacement therapy (ERT) with alglucerase or imiglucerase. The outcomes of interest were the following: growth and development, weight, height, malnutrition, overweight, obesity, basal metabolism, hypermetabolism, insulin resistance, and diabetes. A total of 175 articles were found, of which 28 met the inclusion criteria; these articles were grouped into three central themes: 1) growth of children and adolescents before and after ERT; 2) metabolic changes that remained during ERT; and 3) changes in metabolic status resulting from the treatment.

**Results and discussion:**

The articles included in the present literature review are very heterogeneous, which hinders the analysis of data. They indicated that GD patients usually show low weight and height before ERT, which are improved with treatment in children and adolescents. Studies evaluating the energy metabolism by indirect calorimetry have indicated that the disease is associated with hypermetabolism. In adults, some changes in energy metabolism remain on ERT, and alterations, such as insulin resistance, seem to be associated with the treatment. It is not clear which are the required doses of imiglucerase for obtaining an adequate cost-effective relation, as well as the advisable therapeutic measures to avoid possible long-term adverse effects related to ERT.

**Conclusions:**

ERT tends to normalise the growth of children and adolescents with GD type I, it seems to cause a partial response in relation to some metabolic changes associated with the disease, and it can causes metabolic changes such as weight gain in adult patients. Therefore, additional research is necessary.

## Introduction

Gaucher disease (GD) is an autosomal recessive genetic disease and one of the most common lysosomal diseases. GD results from pathogenic mutations of the *GBA* gene encoding the enzyme glucocerebrosidase (acid β-glucosidase), which is located on chromosome 1q21.31. The absence or low activity of this enzyme leads to a progressive accumulation of its substrate (glucocerebroside) and hence causes the clinical manifestations of the disease [1].

GD is a pan-ethnic disease and its worldwide prevalence is 1 in 50,000-100,000; however, it can be as high as 1 in 850 in individuals of Ashkenazi heritage [2]. The most frequent pathogenic mutations in the *GBA* gene are p.N370S (c.1226A>G) and p.L444P (c.1448T>C) [3]. The clinical variability found in patients with GD is related to the type of mutation in the *GBA* gene and to the proteins, substrates and metabolism of each individual, as well as environmental factors; yet, many of these factors are not completely known.

The medical literature classifies GD into three broad phenotypic categories: a) type I (OMIM #230800), also referred to as adult or non-neuronopathic GD, which is the most common type, characterised by the presence of visceral (hepatosplenomegaly), bone involvement and hematologic symptoms (anaemia and thrombocytopenia), and the absence of significant neurological impairment. Although it is referred to as adult GD, 66% of patients are diagnosed before the age of 20 [4]; b) type II (OMIM #230900), or acute neuronopathic form, has very early clinical manifestations and significant neurological impairment; c) type III (OMIM #231000), or sub-acute neuronopathic form, is similar to type II, but less severe. It is estimated that less than 5% of patients with GD has type II or III GD [5].

For many years, GD was treated with symptomatic therapies and palliative measures such as splenectomy. Since the 90s, however, enzyme replacement therapy (ERT) has been the gold standard treatment. Initially, ERT with alglucerase was used. Afterwards (1998), the emergence of ERT with imiglucerase (Genzyme Corporation, Allston, MA) led to improvements in the quality of life of GD patients [6]. The response to ERT differs according to: a) type of GD (type I or III); b) initial degree of involvement; and c) affected organs. In general, the best response is obtained in the hematological and visceral parameters. Some aspects of the bone disease, such as osteonecrosis, osteofibrosis, and lytic lesions, cannot be reversed. Nevertheless, early initiation of treatment reduces the risk of these irreversible complications [2].

The metabolic status of patients with GD type I has improved with ERT. However, because of its rarity, few large studies have been performed to specifically assess the strength of data highlighting the benefits and limitations of ERT in patients diagnosed in childhood and monitored through adulthood. Therefore, the objective of the present study was to review the literature about the nutritional and metabolic status of patients with GD type I. Our initial hypotheses were: 1) there is high prevalence of low weight and height in non-treated patients; 2) ERT has a beneficial effect on these parameters, which is dose-dependent, but may not completely normalise them; 3) ERT does not fully resolve some of the metabolic disorders of GD; and 4) ERT may as well affect other parameters related to energetic metabolism.

## Material and methods

All steps of this literature review were performed by one of the authors of the present article (DD). Inclusion criteria were established based on: a) type of study: case series (prospective and retrospective, cross-sectional or longitudinal, controlled or not controlled, only if n≥5), clinical trials, systematic reviews, and meta-analyses; b) population: patients with GD type I; c) intervention: ERT with imiglucerase or alglucerase; and d) outcomes: growth and development, weight, height, malnutrition, overweight, obesity, basal metabolism, hypermetabolism, insulin resistance, and diabetes. Studies addressing specific nutritional deficiencies (e.g. iron, vitamin D, and B12) were not included in this review.

The electronic search was performed using Pubmed/Medline and the Scientific Electronic Library Online (scielo.br). Pubmed/Medline was searched using the following search strategy: *Gaucher disease AND (nutrition OR growth OR anthropometry OR malnutrition OR nutritional status OR REE OR resting energy expenditure OR calorimetry OR body composition OR insulin resistance OR type II diabetes OR overweight OR obesity OR calorimetry, indirect OR resting metabolic rate OR energy metabolism OR indirect calorimetry OR basal metabolic rate OR basal metabolism OR metabolic status) NOT Gaucher[Author] Filters activated: Abstract available, Humans, English, French, Italian, Portuguese, Spanish* and studies published until October 2012. As a result, 165 studies were retrieved. The search in *Scielo.br* was performed using the term “*Gaucher disease*” including studies published until October 2012; as a result, 10 additional studies were retrieved.

**Figure 1:**
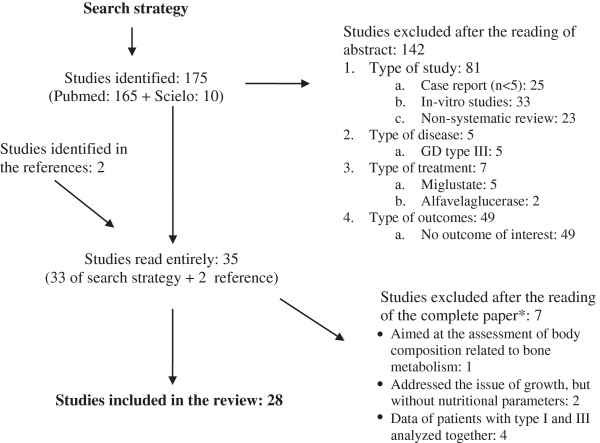
Diagram of the search strategy used in this review (for further details, see section Material and Methods).*did not meet the inclusion criteria.

The abstracts of 175 studies were then reviewed, but only 33 met the inclusion criteria and were analysed (142 were excluded). Based on the review of their bibliographical references, two additional studies [7,8] were identified and included in the present literature review. Thus, 35 articles were read entirely; seven of them were later excluded: although they referred to the nutritional parameter studied, they did not present the specific corresponding data or analysed data from patients with Gaucher type I and III jointly. Twenty-eight papers were included in the present literature review. The description of the search results is shown in Figure 1.

## Results

The 28 studies included in our review were very heterogeneous. In addition, most of them included a small sample and were retrospective and non-controlled (Tables 1 and 2). According to their focus, the selected studies were grouped considering three aspects of the disease: 1) development of children and adolescents during pre- and post-ERT periods (n=17); 2) abnormalities in energy metabolism that remained during ERT (n=10); and 3) abnormalities in metabolic status arising after ERT (n=2, with one of them [9] also providing data related to the aspect of energy metabolism).

**Table 1:** Summary of weight and height in the pre- and post-enzyme replacement therapy (ERT) in children and adolescents with Gaucher disease type I

		**Before enzyme replacement therapy**						**After enzyme replacement therapy**			
**Country**	**Authors**	**Sample <18 years (n)**	**Mean age at diagnosis or presentation (yo)**	**Mean age at baseline or evaluation (yo)**	**Low weight (<p5) (%)**	**Low height (<p5) (%)**	**Growth retardation (%)**	**Sample <18 years at the beginning of ERT (n)**	**Time on ERT (years)**	**Mean dosage of imiglucerase**	**Improved percentile or z-score of height (%)**
Japan	Ida et al. [10]	19	2.7	7.4	10	74	74	-	-	-	-
Canada	MacKenzie et al. [11]	15	-	-	-	-	47	-	-	-	-
ICGG^a^	Kaplan et al. 2006 [12]	496	-	-	-	34	-	-	-	-	-
Tunisia	BenTurkia et al. [13]	11	4.2	8.2	-	-	36	-	-	-	-
Albania	Shehi et al. [8]	8	-	-	-	-	25	-	-	-	-
Israel	Kauli et al. [14]	30	-	5.1	-	-	63^b^	11/30	1-7	30 UI/kg/mo^d^	-^e^
Romania	Grigorescu et al. [15]	6	7.9	14.5	-	33	-	6/6	1.5	45 UI/kg/inf	-^f^
Israel	Zevin et al. [16]	30	5.6	9.9	27	33	33	6/30	1	30 UI/kg/mo	50
Italy	Bembi et al. [17]	6	-	10.2	50	50	-	6/6	1.1	43 UI/kg/mo	50
USA	Kaplan et al. [18]	18	4.5	-	-	50	50	18/18	1	48 UI/kg/inf	71
ICGG^a^	Kaplan et al. [18]	81	6.6	-	-	48	48	36/81	1.5	-	64
Brazil	Oliveira et al. [19]	13	5.8	-	15^c^	36^c^	-	13/13	-	-	55
Argentina	Drelichman et al. [20]	5	5.2	6.2	60	60	80	5/5	1.2	-	80
Brazil	Sobreira; Bruniera [21]	41	-	-	-	46^c^	-	41/41	2	35 UI/kg/inf	77
ICGG^a^	Andersson et al. [22]	702	-	-	-	42	-	702/702	8	39 UI/kg/inf	-^g^

Yo= years old; ICGG= *International Collaborative Gaucher Group.*

a- Anderson et al. 2008 included the patients of the study by Kaplan et al. 2006 and 1996.

b- 63% of patients with growth retardation in childhood (19/30).

c- Less than or equal to score 2 standard deviation.

d- The patients received 2.3 U/kg 3 times weekly, i.e., 30 U/kg/month. In children whose bone disease had worsened, the dose was increased to 30 U/kg every 2 weeks.

e- The authors showed the mean of standard deviation score for the height of the group without the individual patients’ data, but noted that the growth velocity gradually increased.

f -The authors reported that the patients normalised their growth, however did not show the corresponding data.

g -The median height z-score for the study population was −1.4 at baseline and improved to −0.3 after 8 years of ERT, similar to the median for normal population.

**Table 2:** Gaucher disease: summary of the metabolic abnormalities described in treated and untreated patients

**Authors**	**Country**	**Sample**	**Study objective**	**Results**
Corssmit et al. 1995 [23]	The Netherlands	7 untreated patients and 7 healthy controls	Assessing the metabolism of glucose and fatty acids	After continuous infusion of [3-^3^H] glucose, basal glucose production was approximately 30% higher in patients than in controls, while insulin concentration was also slightly higher in patients. The differences in basal glucose production were not associated with the concentration of plasma insulin or other glucoregulatory hormones.
Hollak et al. 1997 [24]	The Netherlands	7 pre- and post-ERT patients and 7 healthy controls	Evaluating the effect of ERT on the clinical and metabolic parameters	Continuation of Corssmit’s study and reassessment of parameters after 6 months of ERT. Increased basal glucose production persisted even with the use of ERT. Patients gained weight in this period.
Langeveld et al. 2007 [25]	The Netherlands	26 pre- and post-ERT patients and 26 healthy controls	Comparing serum adiponectin before and during treatment	GD disease patients showed reduced levels of adiponectin. There was no correlation between the levels of adiponectin, glucose, and insulin. After ERT, the levels of adiponectin increased, but remained below those of the controls.
Langeveld et al. 2008 [26]	The Netherlands	6 patients (3 on ERT and 3 untreated) and 6 healthy controls	Studying the influence of glycosphingolipids storage in the metabolism of glucose and fatty acids.	At basal state, the levels of glucosylceramide and ganglioside GM3 in plasma were higher in patients than in controls. IMGU was lower in patients (n=5) than in controls. The suppression of lipolysis by insulin was observed to be less effective in patients with GD.
Groener et al. 2008 [7]	The Netherlands	Baseline: 27 patients, 15 pre- and post-treatment (13 in ERT and 2 in SRT) and 16 control subjects	Determining the levels of glucosylceramide and ceramide	At basal state, 27 patients were compared with 16 controls and showed glucosylceramide levels greater than three times that of the controls, while the ceramide levels were observed to be slightly lower. Regarding the basal state, patients showed a significant decrease in glucosylceramide levels and a slight increase in ceramide levels A partial normalization of glucosylceramide levels was observed over 72 months.
Ghauharali- van der Vlugt et al. 2008 [27]	The Netherlands	40 treated patients and 30 healthy controls	Determining ganglioside GM3 in plasma	Glucosylceramide and ganglioside GM3 concentrations in plasma were significantly higher than those observed in the controls. Also, the concentrations found for splenectomised patients were higher than those of non-splenectomised patients. In comparison with non-splenectomised patients, the referred concentrations were higher in splenectomised patients. Plasma concentrations of ganglioside GM3 have significantly correlated with plasma chitotriosidase activity, the severity of the disease and hepatomegaly.
Ucar et al. 2009 [9]	Turkey	14 patients undergoing ERT (not overweight) and 14 healthy controls	Assessing insulin resistance in ERT patients (not overweight).	One patient had insulin resistance. The difference between the median glucose of patients (114±5 mg/dL) and that of the post-load controls (103±15.7 mg/dL) was significant. Insulin levels were significantly higher in patients than in controls. Triglycerides and fatty acids were also higher in patients with GD. High insulin levels were positively correlated with free fatty acids, triglycerides, and severity score.

GD- Gaucher disease; ERT- Enzyme Replacement Therapy; IMGU- insulin mediated glucose uptake; SRT- Substrate Reduction Therapy.

### Growth of children and adolescents in the pre- and post-ERT periods

The growth of children and adolescents with GD type I has been monitored by researchers from different countries. They have observed a high prevalence of underweight and reduced height in children and adolescents with GD type I before ERT (Table 1) [8,10-22]. Ten of these studies have compared the basal data with different periods of ERT: when the same study presented data of different times, we considered the data of the first evaluation after the beginning of ERT (Table 1).

One study conducted in South Africa (n=9) analysed the efficacy of low doses of imiglucerase (±10 UI/kg every 2 weeks): weight and height were assessed at the beginning and at the end of the follow-up period. Results showed the number of kilograms and centimetres gained by patients after each year of follow-up: patients grew a mean of 5.6 cm per year and their weight increased significantly over time at a mean of 3.9 kg per year [28].

A study including children with GD in Spain (n=19) associated growth with the levels of insulin-like growth factor (IGF) before and after ERT. Before ERT, there was a positive correlation between the standard deviation of height and that of the IGF-I and IGF binding protein 3 (IGFBP-3). One year after initiating ERT, IGF-I and IGFBP-3 levels were normalised [29].

### Growth and evolution without treatment

Two studies performed in Israel [14,30] assessed growth and pubertal development in treated and untreated patients. The first study found that growth deceleration in untreated patients occurred from 3–5 years old and that height decreased significantly in later childhood. However, at the end of the growth period, the difference between the achieved final height and the target height was not significant, even considering the presence of pubertal development delay in 60% of patients, regardless of the patient being or not on ERT. Delayed growth and puberty were more frequent in patients with more severe disease types [14]. The second study compared mean and standard deviation of height and weight in two groups (untreated: n=34; treated: n=29) at baseline and after three years of follow-up. The mean of z-score of untreated patients at baseline and at the end of the follow-up were respectively: height=−0.39; -0.38; weight=+2.30; +4.83; and the mean of treated patients at baseline and the end of follow-up were: height=−1.18; -0.60; weight=−0.84; -0.25. The authors have not specified the number or percentage of patients with growth retardation [30].

### GD metabolic disorders which remain during ERT

Four studies assessed the basal metabolic rate (BMR) of GD patients by indirect calorimetry. The first of these studies was conducted by Barton et al. [31], who assessed BMR in 25 untreated patients with GD type I and 92 healthy controls. The BMR of patients and controls was compared to the predictive equation of Harris-Benedict. The BMR of controls was 5%, while that of patients was 44% higher than the BMR predicted by the equation. The second study was conducted by Corssmit et al. [23], who assessed BMR in seven untreated adult patients with GD type I and seven healthy controls: patients showed BMR approximately 24% higher than that of controls. In the third study, Hollak et al. [24] continued the study conducted by Corssmit, in which the BMR of the seven patients was reviewed after 6 months of treatment with alglucerase. By comparing the measured BMR values – as predicted by the equation of Harris-Benedict in the pre-treatment period – it was found that they were 29% higher than the expected and, after 6 months of treatment, it remained 20% higher. Finally, in a study involving Brazilian patients, whose mean time of ERT with imiglucerase was 5 years (n=12), it was found that BMR was 27% higher than that of healthy controls [32].

In addition to energy expenditure, other aspects of metabolism were evaluated by other studies, especially regarding glucose metabolism and insulin resistance during pre- and post-treatment periods. A summary of these studies is shown in Table 2[7,9,23-27].

### Abnormalities arising during ERT

A study conducted by Hollak et al. [24] comparing data from pre- and post-ERT periods and involving seven adult patients showed that six of them had gained weight after 6 months of treatment (mean 1.7 kg). Langeveld et al. [33] reported changes in the metabolic status of adult patients undergoing ERT. The study included the follow-up of 42 patients – 35 of them were on ERT – and investigated the relationship between ERT and weight gain, insulin resistance, and type 2 diabetes mellitus (type 2 DM). Before ERT, there were 16% of overweight, the median BMI was 23.3 kg/m^2^, and no case of type 2 DM was found. After ERT was initiated, the median BMI increased to 25.7 kg/m^2^, the prevalence rate of type 2 DM went up to 8.2%, and insulin resistance and overweight rates were respectively 6% and 56%. The untreated patients (n=7) showed initial overweight rate of 14% and, after 8 years, there was a 57% prevalence rate; no cases of insulin resistance or type 2 DM were reported. A study in Turkey evaluated insulin resistance in ERT patients with GD and without overweight (n=14), and showed that they had higher levels of fasting insulin, post-load glucose and insulin when compared to controls. Elevated insulin levels in GD type I patients were positively correlated with free fatty acid, triglyceride, and severity score [9].

## Discussion

The studies found in the present review were very heterogeneous: many analyzed data from patients with GD type I and III, or children/adolescents and adults jointly, for instance. It was therefore necessary to reanalyse the data presented in the original tables focusing only on the outcomes of interest. In some cases, the studies did not show complete data regarding treatment, not including dose, treatment duration, or type of treatment used. In addition, most of them had small sample size and were retrospective and cross-sectional studies, what certainly limited our conclusions.

### Growth of children and adolescents in the pre- and post-ERT periods

The results of the studies were presented in a very different manner: most did not specifically addressed growth-related variables (weight and height), mentioning only one of them (Table 1). Furthermore, several different units of measure were used to show the results: percentile [18], z-score [10,13-15,21,22,30], increase in centimetres or kilograms [28]. Regarding patients' age (Table 1), some researchers collected this variable during the diagnostic period and others during the beginning of the treatment, some used the mean age, whereas others worked with age groups [12,14,22], and others presented tables from which data of interest were collected [11,15-17,20]. Thus, comparisons among the studies could not be made.

The studies showed that untreated children and adolescents had both weight and height below the expected rates for their ages. In addition, when there were early clinical manifestations of the disease, GD was often more severe and growth rates were even more impaired. In general, the studies indicated that ERT had a very positive effect on the growth of children and adolescents, causing a catch-up and a significant improvement in z-score indexes of weight and height. Yet, it was unclear whether the group of patients with GD, as well as their improved indexes, could fully meet the expectations of growth based on their genetic heritage. In this regard, attention should also be devoted to children and adolescents who apparently have a proper growth level, given that it may be below the growth expected for their age when compared to the height of their parents [14,34].

In addition to weight deficit, we also observed that adolescents with GD type I had pubertal development delay [14]. At first, the treatment led to resumption of optimal growth levels and adjustment to the different stages of puberty [34]. It was also suggested that growth retardation could be related to changes in the IGF axis of untreated children and adolescents [29]. Considering the heterogeneity of the disease, it is very important that researches aimed at a better understanding of the factors that interfere with the metabolism of patients continue to be conducted.

The studies did not fully determine the necessary amount of enzyme for the optimum development of children and adolescents: some researchers have shown good results with low doses, whereas others have demonstrated good results with high-dose regimens; however, they have not clarified the severity score and the patients’ age at the beginning of the treatment. Since ERT is an expensive treatment, it is crucial that patients are monitored by a multidisciplinary team – preferably in reference centres, for the adequate identification of the lowest sufficient dose to reverse the current symptoms and prevent possible damages. Furthermore, it is important to point out that the clinical outcome of patients found in studies in the past two decades may have been influenced by other factors besides the treatment with ERT, such as better nutritional intake.

### Growth and evolution without treatment

According to studies conducted in Israel [14,16,30], delayed pubertal development in patients with untreated GD did not interfere with their growth target. Therefore, it seems possible that patients with mild GD may have adequate development without treatment. In agreement with the initial hypotheses and according to all the studies reviewed, we found that: 1) the prevalence of low weight and height is high in untreated patients from different countries; 2) ERT has beneficial effects on these parameters; however, it was not possible to determine whether it can effectively normalise them, since the studies compared the patients’ growth with the population’s growth instead of comparing it with their target of growth based on their parents' height; such evaluation was only assessed in the study by Kauli et al. [14]; 3) some patients could reach their target growth even without treatment [14,16,30]; and 4) in relation to the dose required for adequate development, there seems not to be a consensus on the fact that higher doses are more effective in solving growth problems: one study has found good results with low doses [28], whereas another one has showed that the results are dose-dependent and more severe patients require a greater amount of enzyme [18]. Further studies may clarify this aspect of growth in patients with GD type I.

### Metabolic disorders of GD that ERT cannot revert

Studies evaluating the energy metabolism of patients using indirect calorimetry have suggested that the disease causes hypermetabolism [23,24,31,32], which would be responsible for inadequate growth in untreated children and adolescents. At first, this hypermetabolism is not observed in other lysosomal diseases with hepatosplenomegaly [31]. After 6 months of ERT, a small decrease in energy expenditure was observed in the seven patients evaluated before and after ERT [24]. However, these patients with BMR 30% higher than the one estimated by the equation remained with 20% above the expected. This result is consistent with that found by Doneda et al. [32], whose patients, continued to have higher BMR than their controls after 5 years of ERT with imiglucerase, hence indicating that the treatment could not normalise the energy expenditure of patients with GD. Langeveld et al. [35] evaluated whether the hypermetabolism of patients participating in the research conducted by Corssmit [23] and Hollak [24] was associated with thyroid disease; no associations were found between these variables. Despite some hypothesis on the relation between hypermetabolism and GD, it remains unclear.

Patients with GD showed impaired glucose metabolism [23,24]. In addition, when compared to controls, the following changes were found in GD patients undergoing ERT: lower plasma adiponectin levels [25], higher levels of glucosylceramide in patients than in baseline controls [7,26], partial normalisation of glucosylceramide levels after 72 months of treatment [7], higher levels of post-load glucose, insulin, triglycerides, and fatty acids [9], and greater concentration of ganglioside GM3 in plasma [26,27]. These changes have indicated that, despite the efficacy of the treatment in solving some manifestations of the disease, it has not been as effective in treating certain metabolic changes. The hypothesis proposed in the present review that ERT shows limitations in solving some GD metabolic problems seems to be corroborated by the studies analysed; therefore, further studies and longer follow-up periods may contribute to better understanding both the mechanism of the disease and its evolution.

### Abnormalities associated with ERT: overweight, insulin resistance, and type 2 DM

A study conducted by Langeveld et al. [33] in the Netherlands, with a cohort of 49 patients, identified a higher percentage of patients with type 2 DM and overweight after some years of ERT. The development of type 2 DM may be related to the several changes found in the glucose metabolism of patients with GD.

The prevalence of overweight was not different from that observed in studies involving the general population; yet, overweight onset was quite different: GD patients started presenting lower levels of weight when ERT was initiated (even considering organomegalies). Hence, weight gain was more significant than in the general population. According to the author, one of the reasons for this finding was that ERT reduced the hypermetabolism of GD, but without the proportional adjustment of energy intake by patients, thus resulting in a higher level of weight gain. Regarding children and adolescents, this caloric difference would be directed to growth and would explain why they have not shown the same trend of overweight observed in adults undergoing ERT.

According to a study conducted by Ucar et al. [9], it is possible that patients with more severe GD have a reduction in insulin sensibility and a greater tendency to develop type 2 DM, even without being overweight. Additional studies with larger numbers of patients and other populations are needed to verify this hypothesis.

Another consideration relates to differences in parameters due to age at the beginning of treatment: it is possible that the adverse effects observed in patients who started ERT in an older age are different from those who started treatment at an early age. There is no consensus about the dose required to achieve a cost-effective adequate and therapeutic measures advisable to avoid possible long-term adverse effects, such as weight gain and insulin resistance, as described in some studies. Currently, this information is even more relevant as new enzymes have been approved, and it is important that doctors and patients have consistent information about each of them in order to choose the best treatment.

## Conclusions

Most studies have not specifically addressed growth and metabolism and GD. Nevertheless, several studies have dealt with this topic, especially regarding the growth retardation rate of children and adolescents and the delayed puberty in untreated adolescents. Although some changes in metabolism remained, ERT contributed to normalise the development of children and adolescents. Studies have also shown that some patients with milder disease may have adequate growth without treatment. As for adults, ERT seemingly causes weight gain, insulin resistance, and development of type 2 DM.

According to the available literature, it is not possible to determine if hypermetabolism is a characteristic of GD, if it is caused by factors also found in other chronic diseases, or even if it correlates with the severity of the clinical manifestations of patients, which has an impact on the establishment of the prognosis or management. We believe that further studies, especially controlled and longitudinal studies including an adequate sample size, are needed to address these gaps in knowledge.

## Abbreviations

GD: Gaucher disease; Type 2 DM: Type 2 Diabetes Mellitus; BMR: Basal Metabolic Rate; BMI: Body Mass Index; ERT: Enzyme Replacement Therapy; ICGG: International Collaborative Gaucher Group Registry.

## Competing interest

All authors confirmed that there are no conflicts of interests to declare.

## Authors’ contributions

DD developed the search strategy, reviewed the articles, and drafted the manuscript. CCM, CBN read the manuscript and suggested changes to its organisation. IVDS supervised all stages of research, presentation, and revision of the manuscript. All authors read and approved the final manuscript.
